# Turbulence causes kinematic and behavioural adjustments in a flapping flier

**DOI:** 10.1098/rsif.2023.0591

**Published:** 2024-03-20

**Authors:** Emmanouil Lempidakis, Andrew N. Ross, Michael Quetting, Krishnamoorthy Krishnan, Baptiste Garde, Martin Wikelski, Emily L. C. Shepard

**Affiliations:** ^1^ Biosciences, Swansea University, Singleton Park, Swansea SA2 8PP, UK; ^2^ School of Earth and Environment, University of Leeds, Leeds, UK; ^3^ Max Planck Institute of Animal Behavior, Radolfzell, Germany; ^4^ Centre for the Advanced Study of Collective Behaviour, University of Konstanz, Konstanz, Germany

**Keywords:** freestream turbulence, stability, wing kinematics, flight cost, flapping flight

## Abstract

Turbulence is a widespread phenomenon in the natural world, but its influence on flapping fliers remains little studied. We assessed how freestream turbulence affected the kinematics, flight effort and track properties of homing pigeons (*Columba livia*), using the fine-scale variations in flight height as a proxy for turbulence levels. Birds showed a small increase in their wingbeat amplitude with increasing turbulence (similar to laboratory studies), but this was accompanied by a reduction in mean wingbeat frequency, such that their flapping wing speed remained the same. Mean kinematic responses to turbulence may therefore enable birds to increase their stability without a reduction in propulsive efficiency. Nonetheless, the most marked response to turbulence was an increase in the variability of wingbeat frequency and amplitude. These stroke-to-stroke changes in kinematics provide instantaneous compensation for turbulence. They will also increase flight costs. Yet pigeons only made small adjustments to their flight altitude, likely resulting in little change in exposure to strong convective turbulence. Responses to turbulence were therefore distinct from responses to wind, with the costs of high turbulence being levied through an increase in the variability of their kinematics and airspeed. This highlights the value of investigating the variability in flight parameters in free-living animals.

## 1. Introduction

The impacts of atmospheric turbulence are well-established in the aviation industry (e.g. [1,2]). Turbulence can increase drag [3], resulting in reduced lift, sudden drops in altitude and instability. Consequently, pilots modify their flight route and/or altitude to avoid highly turbulent regions in the atmosphere. This helps prevent potential aircraft damage and reduces fuel wastage, although the detours themselves can be associated with a substantial cost [4]. Fluctuations in the wind, or gustiness, should also have an important impact on animal flight [5]. Yet how animals respond to this element of turbulence in the wild, and the implications for energy expenditure and route selection, remain little studied (though see [6]).

Experiments in the laboratory (e.g. [7]) and small natural habitat patches [8,9] have shown that insects and hummingbirds adjust their kinematics to enhance flight stability and control when exposed to simulated turbulence. This in turn, can lead to increased flight costs [10]. For example, wild orchid bees (*Euglossa imperialis*) extended their legs in turbulent conditions, leading to a 30% increase in power output [8]. Likewise, ruby-throated hummingbirds (*Archilochus colubris*) subjected to turbulence in a wind tunnel, made fine-scale adjustments to their body and tail and increased their stroke amplitude to retain a relatively stable position. This also resulted in higher drag [8]. Anna's hummingbirds (*Calypte anna*) varied their wingbeat frequency and amplitude when flying in von Kármán vortex streets, and their metabolic costs increased up to 25% as the scale of turbulence increased [5]. The turbulence length scale was also important for bumblebees (*Bombus impatiens*, *Bombus terrestris*), as the rolling instabilities were highest when the length scales were similar to their wingspan [11–13]. Animals can therefore alter their mean postural and kinematic parameters in response to turbulence, or the variability in these parameters, and both can have energetic consequences.

However, significant differences exist between laboratory conditions and real-world settings in terms of the turbulence experienced by animals. In laboratory studies, turbulence is often simulated by introducing structures like cylinders into the flow, creating discrete, alternating vortices that are shed at predictable intervals. These von Kármán vortex streets are seldom found in nature. By contrast, free-stream turbulence is characterized by rapid, intricate and unpredictable disturbances in the flow, spanning a wide range of spatial and temporal scales [10]. Free-stream turbulence can be more accurately simulated by introducing a grid into the flow [10], although here the scale of turbulence is limited, being determined by the dimensions of the grid. In the laboratory, hummingbirds modified their flight kinematics to enhance flight stability in the presence of both turbulence forms. However, their specific kinematic responses varied depending on whether the turbulence was generated by a grid or a cylinder [10].

Another difference between simulated turbulence and free-stream turbulence is the possibility of strong upward components in the latter, driven by thermal convection and/or wind–terrain interactions. Soaring birds have evolved tactics to take advantage of rising air currents e.g. adjusting their flight path to gain altitude in updrafts (e.g. [14,15]). While circling within thermal updrafts to gain altitude tends to be inefficient for most flapping fliers [16], they may be able to extract energy in other ways. For instance, European bee-eaters (*Merops apiaster*) transitioned from flapping to soaring/gliding flight when turbulence intensity surpassed a specific threshold [17]. Anecdotal observations from other flapping fliers, like pigeons soaring over buildings in strong wind conditions, also indicate their ability to take advantage of the upward component of turbulence in certain situations. Therefore, whether turbulence incurs a net cost for flapping fliers should depend on their need for heightened flight control.

In this study, we fitted solo-flying pigeons with high-frequency data-loggers and released them in varying levels of turbulence. We have previously shown that the fine-scale vertical displacements of homing pigeons (*Columba livia*) perform well as a proxy for turbulence in the natural environment [18]. We now use this approach to examine flight responses to free-stream turbulence, estimated along the flight paths, and resolved to a spatial scale of a few hundred metres [18]. Our objectives were to: (1) assess how birds maintain flight control in increasing turbulence, specifically whether they vary their mean wingbeat frequency and amplitude and/or make stroke-to-stroke changes in their kinematics [10], and (2) investigate how changes in mean wingbeat frequency and amplitude affect propulsive efficiency. The kinematic responses and power requirements are likely to underpin behavioural responses to turbulence, which we address in a further objective, (3) to assess how birds adjust their airspeed and flight altitude, expecting that if turbulence represented a net cost, pigeons would fly faster and at lower altitudes to reduce the level of turbulence they were exposed to [19]. Taken together this should provide insight into the significance of turbulence on flapping fliers, and how it compares to wind, where the impact is well known.

## 2. Methods

### 2.1. Animal movement data

Homing pigeons are an excellent model species for studying the impact of turbulence on flapping flight since they consistently return to their loft in various conditions, following a well-known route once they become familiar with the release location [20,21]. Ten individual homing pigeons (*Columba livia Linnaeus*) (maximum number of individuals available) weighing between 442 and 476 g were released in an agricultural area near Radolfzell, Germany, with the objective of returning to their loft [18]. Solo flights ensured that kinematic parameters were not influenced by proximity to conspecifics [22]. To ensure the flight trajectories and kinematics were not influenced by route familiarity, each bird was released over 30 times before data collection commenced [23]. On any given day, up to six birds were randomly chosen for release based on their individual identifier and the order of the previous release days of the same field trip, resulting in a total of 23 days and 124 releases. We ensured that birds flew in a wide range of turbulence conditions by varying the time of release (beginning either early or late morning) and the season, collecting data over two summers (July 2018, 2019, 76 flights) and one spring (April 2019, 48 flights).

Birds were fitted with a combined ‘Daily Diary' (DD) tag from Wildbyte Technologies, Swansea University, UK, along with a GPS logger (GiPSy 5) provided by Technosmart Europe, Guidonia-Montecelio, Italy. Both devices were integrated into a single three-dimensional printed housing [24]. The DD units logged triaxial acceleration at 200 Hz and barometric pressure at 20 Hz. The GPS logger recorded data at a sampling rate of 1 Hz for all flights, except for the flights conducted in April 2019, which were initially sampled at 5 Hz and later subsampled to 1 Hz for consistency. Each DD logger was connected to a GPS unit and programmed to take the time stamp from the GPS. Wind speed and direction were evaluated using the hourly u and v wind components retrieved from the ERA5 global reanalysis [25].

### 2.2. Assessing turbulence

In a previous study, we have shown that fine-scale changes in pigeon flight height perform well as a qualitative proxy of atmospheric turbulence levels, with flight height becoming more variable as turbulence increases [18]. Specifically, an ultralight (ATOS VRS280, www.a-i-r.de) equipped with a triaxial ultrasonic anemometer (uSonic-3 CLASS A) flew a stable course behind pigeons undertaking short homing flights. Using the root mean square of the three wind velocity components, we estimated the log power spectrum and fitted the ideal −5/3 power law that turbulence is expected to follow over the inertial subrange of frequencies. The constant of proportionality of the power law fit provided us with a qualitative measure of the turbulence present during the flight (for details see Lempidakis *et al*. [18]). We tested different metrics derived from sensors onboard the birds, for their ability to predict changing turbulence levels and found that the interquartile range of pressure fluctuations was the best performing proxy (for details see Lempidakis *et al.* [18]). Barometric pressure sensors were positioned face-down within the tag housing, such that they were not exposed to variation in dynamic pressure arising from changes in airspeed. The main variation in pressure was therefore related to changes in flight height.

In this study, we use this proxy to predict the turbulence experienced by the birds over each 15 s segment of flight. The 15 s window length was selected after trials showed that longer segments gave similar results whereas shorter segments produced a number of negative turbulence estimates. This interval was also considered sufficient to capture the scale of large eddies at the mean flight altitude above ground (approx. 80 m), while also considering the mean flight ground speed (around 20 m s^−1^). Dividing flights into 15 s segments resulted in several shorter segments at the end of the flights. These were excluded from the analyses if they were less than 10 s, which led to the exclusion of 43 segments.

We evaluated whether pigeons flew under high or low turbulence levels, relative to those recorded at the study site within a given season. As in [18], we estimated convective (w*) and shear velocity (u*), as proxies of thermal and mechanical turbulence, respectively, using the ERA5 global reanalysis [26]. The shear velocity (u*) depends on the surface shear stress and gives a velocity scale for mechanical (or shear-generated) turbulence, while the convective velocity (w*) depends on the surface heat flux and so provides a velocity scale for thermal (or convective) turbulence. These quantities are commonly used in the atmospheric turbulence literature to provide velocity scales for the magnitude of turbulence [19] and, although they do not provide the fine spatial or temporal resolution of the pressure-based turbulence proxy, they have the advantage of being readily calculated over a long time period from the reanalysis dataset. They also allow us to put our qualitative turbulence proxy values into a climatological context. We used *t*-tests to assess whether w* and u* during pigeon flights were different to those experienced during July–August (for the July releases) and April (for the April releases) for the period 2016–2022 (± 4 h from the earliest and the latest flight in each period).

### 2.3. Data processing (i) the impact of turbulence on flight kinematics and effort

Acceleration data were corrected for minor differences in tag orientation between deployments [27] by selecting a segment of level flight of at least 2 s and then adjusting the acceleration data so that the gravitational acceleration in the heave axis (dorsoventral acceleration) was equal to 1*g*. Corrections were applied to each flight using DDMT (Daily Diary Multiple Trace, version: 25 November 2022, Wildbyte Technologies, GitHub).

We identified individual wingbeats following [28]. In summary, we smoothed the raw heave values over 20 events to remove the high frequency noise from the heave signal. We then calculated the rate of change of the smoothed heave values over 20 events (trials showed this period performed best in identifying wingbeat peaks). Peaks were identified as positive-to-negative transitions in these differential values, once a filter had been applied to remove high frequency oscillations that would be identified as false peaks. The threshold for this filter was adjusted for individual flights, ensuring that the positive-to-negative transitions identified clear peaks that were associated with wingbeats. This process was completed in DDMT software package (see manual pages 60–64 for more details at GitHub). Finally, the wingbeat frequency was estimated from the period between consecutive peaks in the R programming language [29].

We used the amplitude of the heave acceleration as a proxy for wingbeat amplitude [28]. This approach is based on the correlation between body acceleration signal and wing displacement within a wingbeat cycle. In a previous study we found a linear relationship between the heave amplitude and the wingbeat amplitude. From here on, the term ‘wingbeat amplitude' therefore refers to the amplitude of the heave signal. The heave amplitude was calculated as the difference between the peak and the trough of the raw heave values within a single wingbeat cycle [28].

Two types of kinematic response have been found for birds flying in turbulence in controlled conditions: an increase in mean wingbeat frequency or amplitude, and stroke-to-stroke variation in kinematics [10]. We therefore quantified the mean wingbeat frequency and amplitude per segment and used the standard deviation in these parameters to assess the kinematic variability.

We assessed the implications of changes in mean wingbeat frequency and amplitude for power output by calculating the flapping wing speed, which is a product of the mean wingbeat frequency and amplitude per segment [30] (here taken as the amplitude of the raw heave acceleration multiplied by the wingbeat frequency). We calculated this separately for ascending and descending flight, using the climb rate (*Vz*, see below), for ascents (*Vz* > 0) and descents (*Vz* < 0).

### 2.4. Data processing (ii) the impact of turbulence on track tortuosity, airspeed and flight height

We investigated the influence of turbulence on fine-scale track tortuosity by analysing the turning angle, corresponding to the difference in flight heading between consecutive pairs of GPS fixes (at 1 Hz with circular scale: −180° to 180°). The circular standard deviation in turning angle served as a measure of horizontal tortuosity per segment. We opted for this method over the straightness index (SI) due to the relatively short segment length (mean = 280 m), which limited the inter-segment variation in SI. All attributes and calculations were conducted using the move package, version 4.0.0 [31].

Airspeed and headwind component (HWC) were determined by assessing the angle between the GPS heading/ground speed vector and the wind speed vector [32]. A positive HWC indicated flight with a headwind and a negative HWC flight with a tailwind [33]. Wind speeds and directions estimated using ERA5 were compared with measurements made by the anemometer stationed at the release site (10 s sampling frequency) to verify that the prevailing conditions from the coarser ERA5 estimates were in good agreement with the local conditions captured by the anemometer. As the anemometer did not record conditions for three flights, ERA5 was selected for analysis.

We examined how turbulence affected flight height (vertical route choice), with the expectation that pigeons would opt for lower altitudes to avoid altitudes with high turbulence, similar to the response to headwinds [34]. Flight height above sea level (ASL) was determined by analysing the barometric pressure data [35] recorded by the DD tags and smoothed over 2 s. Changes in sea level pressure were factored in prior to processing each flight using the barometric pressure values recorded at the release site. Using altitude ASL, we estimated the climb rate per second (Vz, m s^–1^). Finally, altitude ASL was converted to altitude above ground level (AGL) by subtracting the elevation of the terrain acquired from a 30 m digital surface model (DSM) (source).

### 2.5. Statistical analysis

We assessed the impact of turbulence on various flight characteristics, using a multivariate generalized additive mixed effect model (GAMM) from the ‘mgcv' package version 1.8.31 (source) [36], with the family set as ‘mvn'. This approach allowed us to assess the importance of turbulence in relation to other covariates at two levels: within and between response variables (sub-models).

Our multivariate model included eight response variables (sub-models), each of which was assessed per flight segment: (1) mean flight altitude, (2) mean flight tortuosity (the standard deviation of GPS turning angle), (3) mean wingbeat frequency, (4) mean wingbeat amplitude, (5) mean airspeed, (6) standard deviation of wingbeat frequency, (7) standard deviation of wingbeat amplitude and (8) standard deviation of airspeed. Turbulence and HWC were included as covariates for all response variables. Climb rate and mean flight altitude were included as covariates in the sub-models of mean and standard deviation of wingbeat frequency, amplitude, and airspeed. This allowed us to account for the influence of ascents/descents and flight altitude on kinematics and airspeed. We ran the same multivariate model with the response variables standardized (scaled and centred) to examine the size of the effects of all covariates on the same scale.

We assessed the distribution of each covariate and square root transformed turbulence and flight altitude above ground to improve model fitting. In all sub-models, flight ID was included as a random factor to account for other effects that might have had an influence during each individual flight (i.e. the presence of raptors in the area). The basis dimension ‘k' was optimized using the option ‘select = TRUE'. To address temporal and spatial autocorrelation, we incorporated the time and the longitude/latitude of the mid-point of each segment nested within the pigeon ID (after testing) using the corARMA and corSpatial functions from the ‘nlme' package version 3.1.148, [37]. We also transformed response variables using the best transformation identified with the ‘boxcox' function (see electronic supplementary material, table S1) (package: ‘MASS' version 7.3.58.1) [38] (RRID:SCR_019125) where necessary to yield satisfactory model residuals. Model residuals were visualized and inspected using ‘gam.check' function (package: ‘mgcv') and autocorrelation function (acf, package: ‘stats', [38]).

In order to quantify the magnitude of the effect of turbulence on the different response variables, we assessed the size of the partial effect of turbulence in the un-standardized multivariate GAMM at the minimum and maximum turbulence levels. We then present the overall range observed in the response variable, and the percentage of the partial effect in relation to this range that is seen at the minimum and maximum turbulence levels. The other option would have been to use the GAMM model to predict the response variables by varying turbulence while keeping other predictors constant (summed effect). We chose not to use this approach as the effect of turbulence is sensitive to the fixed values selected for other predictors. Statistical analyses were carried out using RStudio version 1.2.5 (RStudio Team, 2015) (RRID:SCR_000432) and the R programming language version 4.2.1 [29].

## 3. Results

**Figure 1.**
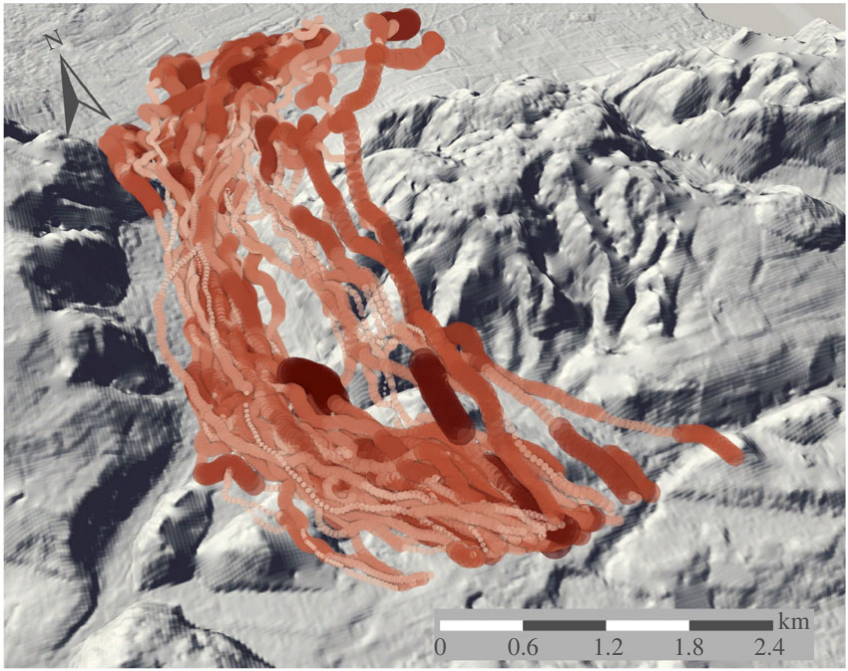
Pigeon GPS flight tracks (red) in the study site with predicted turbulence. Increasing symbol size and darker colour indicate higher turbulence.

After excluding flights with a damaged GPS or DD logger, flights with a landing break and two non-solo flights, 61 flights remained. These were subdivided into 1067 segments across seasons (excluding segments shorter than 10 s) (figure 1).

### 3.1. Sampled turbulence in relation to seasonal trends in the study area

**Figure 2.**
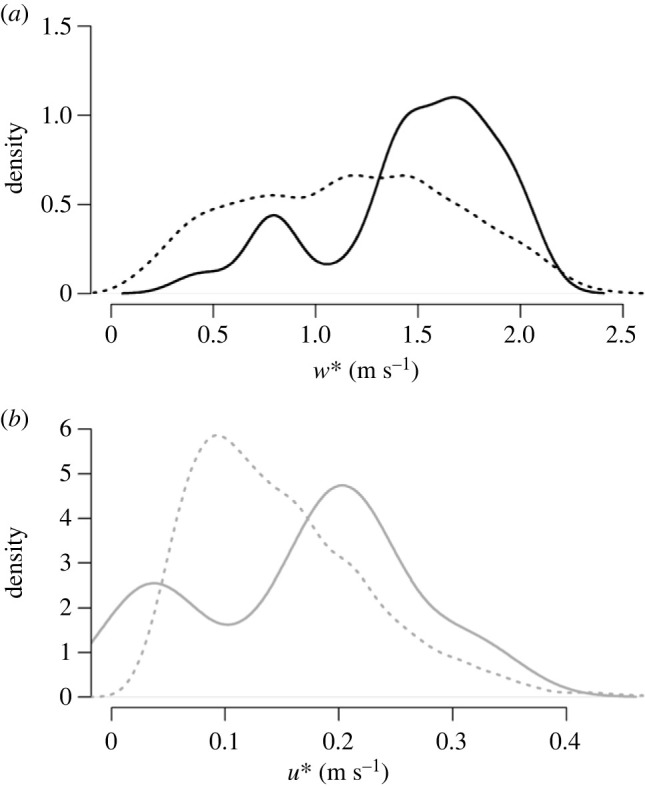
Comparison of the density distribution of (*a*) convective (w*) and (*b*) shear velocity (u*) during 61 pigeon flights (solid lines) and all values estimated for July–August from 2016 to 2022 (dashed lines). Both w* and u* were estimated with data from the ERA5 global reanalysis.

Mean convective velocity (w*) during the July pigeon releases was statistically higher than the overall mean w* for July–August over the last 7 years (2016–2022, figure 2*a*) (two sample *t*-test: *t* = −3.3565, d.f. = 5186, *p*-value = 0.0004). In the April releases, the mean w* was not statistically different from the mean April conditions (two sample *t*-test: *t* = 1.3892, d.f. = 1865, *p*-value = 0.1649). Mean values of u* during releases were not statistically different from the overall mean for July (figure 2*b*, two sample *t*-test: *t* = −1.328, d.f. = 5186, *p*-value = 0.1842) or April (two sample *t*-test: *t* = −0.23804, d.f. = 1865, *p*-value = 0.8119). The maximum levels of w* and u* that pigeons experienced were greater than the 80th centile of those available in both seasons. The July releases therefore occurred on days with strong convective turbulence for the season, whereas the April days were representative of the climatology in terms of mean turbulence. In both months, we sampled days with high mechanical and convective turbulence (top 20% of available turbulence levels).

### 3.2. Multivariate model results

**Figure 3.**
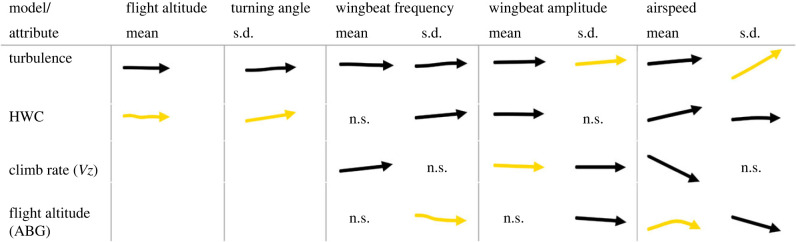
Summary of the standardized multivariate GAMM sub-models and effects (deviance explained: 75.8%, *n* = 1067). The scaled and centred response variables allow the effects to be compared between different responses (columns). Arrows pointing upwards or downwards indicate positive or negative effects on the response, respectively. The slope of the arrows indicates the magnitude of the effect with a steeper slope indicating a larger effect. The composite of sub-models was created using the exact outputs of the GAMM models. Non-significant effects are indicated as n.s., while the largest effect in each sub-model is highlighted in yellow.

All flight variables were significantly influenced by turbulence. The largest effects were on wing kinematics. Here, turbulence was the most important predictor of the SD in wingbeat frequency and amplitude. Mean frequency and amplitude were most affected by changes in climb rate. Nonetheless, turbulence still had a significant effect that was comparable to that of the HWC in its effect size (figure 3; electronic supplementary material, figure S1). Turbulence was also the main predictor of the variability in airspeed. A detailed description of each of the model outputs is provided below.

### 3.3. Flight kinematics and efficiency

Turbulence had opposite effects on the mean wingbeat frequency and amplitude (figure 3; electronic supplementary material, figures S2, S4), but frequency and amplitude were only very weakly correlated (Pearson correlation test: coefficient = 0.09, *t* = 2.8864, *p*-value = 0.004). At the maximum level of turbulence, wingbeat frequency decreased by −2.1% of its range (*p*-value = 3.40 × 10^−04^) and wingbeat amplitude increased by 1.7% of its range (*p*-value = 1.15 × 10^−03^) (table 1). Birds made similar kinematic adjustments in relation to climb rate, with higher wingbeat frequencies (*p*-value = 2.00 × 10^−16^) and lower amplitudes (*p*-value = 1.85 × 10^−06^) during climbing (figure 3; electronic supplementary material, figures S2, S4). Wingbeat frequency tended to be higher at lower altitudes (although it was not significant in the standardized model, electronic supplementary material, tables S2, S3), but amplitude did not change with height (electronic supplementary material, tables S2, S3 and figures S2, S4). There was no indication of any temporal variation in the mean wing kinematics throughout the study period (electronic supplementary material, figure S6).

**Table 1.** The percentage change of the effect of turbulence in the GAMM multivariate model in relation to the range of the response variable.

response	range of response	change at maximum turbulence (%)	change at minimum turbulence (%)
mean wingbeat frequency (Hz)	3.0	−2.1	1.6
SD of wingbeat frequency (Hz)	4.6	8.8	−11.4
mean wingbeat amplitude (*g*)	5.6	1.7	−1.9
SD of wingbeat amplitude (*g*)	4.6	11.0	−12.0
mean airspeed (m s^−1^)	14.1	3.1	−5.4
SD of airspeed (m s^−1^)	4.6	23.9	−24.0
flight altitude (m)	321.6	−1.7	2.0
turning angle (°)	30.9	4.8	−3.4

When the wingbeat frequency and amplitude were considered together, turbulence had no effect on the flapping wing speed (see methods: data processing (i) the impact of turbulence on flight kinematics and effort), either during ascending (simple linear regression: *p*-value = 0.381, estimate = 0.16, d.f. = 595) or descending flight (simple linear regression: *p*-value = 0.444, estimate = 0.183, d.f. = 468) (electronic supplementary material, figure S7).

**Figure 4.**
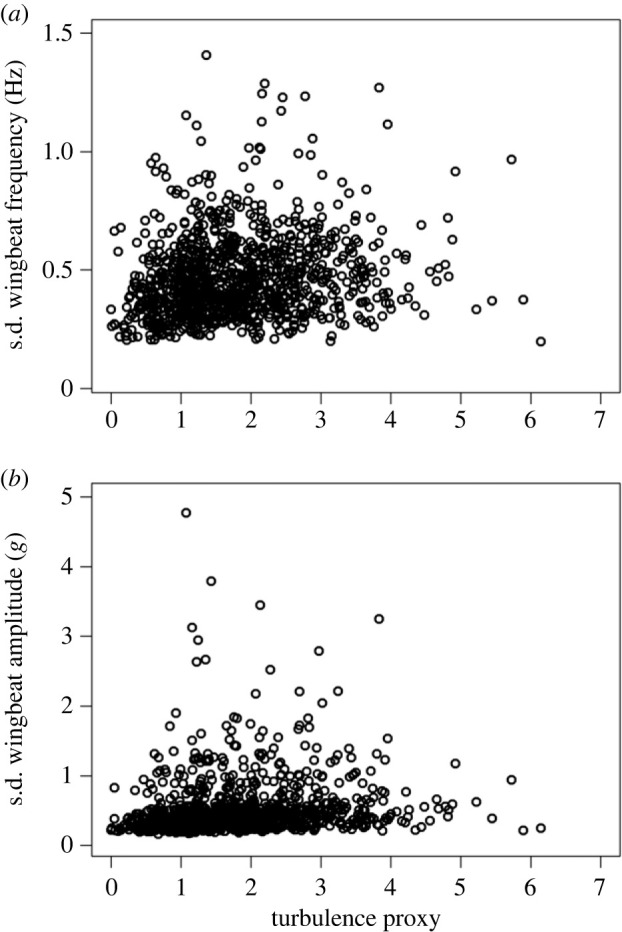
The standard deviation of (*a*) wingbeat frequency and (*b*) amplitude per segment. Both standard deviations increased with turbulence with frequency showing a clearer trend. The amplitude of the heave acceleration was adopted as a proxy to quantify wingbeat amplitude in *g*.

Turbulence had the most significant impact on increasing the standard deviation of wingbeat frequency and amplitude (figure 4) (*p*-value ≤ 2.00 × 10^−16^, in both cases) (electronic supplementary material, figures S3, S5) with the variability in frequency and amplitude increasing by 8.8% and 11% at maximum turbulence levels, respectively (table 1). Both variables also increased (but to a lesser degree) as birds flew closer to the ground (*p*-value = 1.64 × 10^+03^, *p*-value = 1.42 × 10^+03^, respectively) (electronic supplementary material, figures S3, S5). However, HWC only had a significant effect on SD frequency (*p*-value = 2.00 × 10^−16^) and climb rate only on SD amplitude in the standardized model (*p*-value = 1.13 × 10^−02^) (figure 3; electronic supplementary material, tables S2, S3).

### 3.4. Flight speed and route adjustments

The most influential predictor of horizontal path tortuosity (range: 2.3–33.7°, with a mean of 8.3°) was the HWC (*p*-value ≤ 2.00 × 10^−16^), with tortuosity (see methods: Data processing (ii) the impact of turbulence on track tortuosity, airspeed and flight height) increasing with the HWC (increase of 1.7° per segment) (electronic supplementary material, figures S3, S5). Segments also became more tortuous with increasing turbulence strength (*p*-value = −8.53 × 10^−07^), with an increase of 4.8% of the range in standard deviation at the maximum turbulence level (table 1).

Flight height above ground (mean per segment 79.3 m, range 1.5–329.4 m) was most affected by the HWC (*p*-value ≤ 2.00 × 10^−16^) decreasing by approximately 0.7 m with a HWC change from −2 to −1 m s^−1^. Birds therefore flew lower when flying into headwinds and higher when encountering tailwinds (negative HWC) (electronic supplementary material, figures S2, S4). Flight height also showed a small but significant decrease with turbulence (1.7% of the range in flight height at the maximum turbulence level) (*p*-value = 4.85 × 10^−02^, 0.35 m from minimum to maximum turbulence, electronic supplementary material, figures S2, S4 and table 1). The mean flight height in low turbulence segments (turbulence ≤1.1, 25% quartile) was also different to that of high turbulence segments (turbulence ≥ 2.4, 75% quartile) (83.6 and 80.2 m, respectively, Wilcoxon test, *V* = 0, *p*-value < 2.2 × 10^−16^).

The most important determinant of airspeed (range: 12.8–26.9 m s^−1^, mean = 19.1 m s^−1^) was the climb rate (*p*-value ≤ 2.00 × 10^−16^), with airspeed decreasing by more than 6 m s^−1^ across segments with climb rates of –3.6 to 2.4 (mean = 0.06) m s^−1^ (figure 3; electronic supplementary material, figure S4). However, birds increased their airspeed with both headwind (*p*-value ≤ 2.00 × 10^−16^) and turbulence (*p*-value = 3.59 × 10^−05^) (figure 3; electronic supplementary material, figures S2, S4). Although the effect size for turbulence was relatively low with an increase of approximately 1 m s^−1^ from lowest to highest turbulence, compared to an increase of approximately 3 m s^−1^ from highest tailwind to highest headwind (electronic supplementary material, figure S4).

Conversely, turbulence emerged as the most significant predictor influencing airspeed variability (*p*-value ≤ 2.00 × 10^−16^), with the variability in airspeed increasing as turbulence intensified (23.9% at maximum turbulence, table 1) and flights were conducted closer to the ground (*p*-value = 3.09 × 10^−04^) (figure 3; electronic supplementary material, figures S3 and S5).

## 4. Discussion

We examined the impact of turbulence on free-flying birds in the field and found that it had a significant effect on all kinematic and behavioural responses. Our pigeon-based proxy for turbulence allowed us to examine how it impacted animals at fine-scales compared to what has been possible in the field to date [17,39]. The marked variation in turbulence that we observed along each flight track (mean distance 5.2 km) supports the need for high resolution proxies, indeed, in some cases turbulence varied as much within flights as it did between them (figure 1). Nonetheless, we were still restricted to assessing the mean response over 15 s flight segments (approx. 280 m) and the scale and location of the segments will inevitably introduce error into our characterization of turbulence responses.

### 4.1. Kinematic responses to turbulence

Flapping fliers can mitigate the impact of turbulence by increasing their mean wingbeat frequency and amplitude. This enhances manoeuvrability and stability in relation to external perturbations by increasing the rate at which discrete control inputs are applied [10,40,41]. It was therefore interesting that pigeons in this study reduced their mean wingbeat frequency with increasing turbulence, although the change per segment was small. This contrasts with the response of ruby-throated hummingbirds, which showed a small but significant increase in wingbeat frequency when flying in strong freestream turbulence generated by a moving grid [10]. However, both pigeons and hummingbirds increased their mean wingbeat amplitude in highly turbulent flow, which may reduce the need for instantaneous compensation, i.e. adjustments made from one wingbeat cycle to the next (see below).

We observed the same combined response of a decrease in wingbeat frequency and an increase in wingbeat amplitude in climbing flight, which is a power intensive mode of flight. Nonetheless, when mean wingbeat frequency and amplitude are considered together, we find no evidence for a net change in propulsive efficiency, as the flapping wing speed did not vary with turbulence. Consequently, any changes in the aerodynamic forces due to relative flow velocity over the wing would arise from the contribution of inertial effects, i.e. variation in forward speed, or more variable wingbeat kinematics. We did find a small increase in airspeed (1 m s^−1^ across turbulence levels or 3.1% of the range in airspeed), which is therefore achieved without a measurable increase in the aerodynamic forces produced by the bird, raising the intriguing possibility that birds could be increasing their speed by extracting energy from the environment.

Overall, our data indicate that turbulence represents an energetic cost rather than benefit, due to the marked increase in the variability of their kinematics, as well as flight speed. Similar to hummingbirds, which displayed increased kinematic variability when flying in both freestream turbulence and von Kármán vortex streets [5,10], the most pronounced response to turbulence was an increase in the pigeons' kinematic variability, specifically in the standard deviation of wingbeat frequency and amplitude (8.8% and 11%, respectively). Pigeons therefore appear to vary their stroke-to-stroke kinematics [10] to compensate for turbulence to a greater degree than adjusting their mean kinematics. The marked increase in kinematic variability in hummingbirds has been linked to a substantial increase in energy expenditure [5,10]. The same is likely to be true for pigeons, given the energetic implications of intermittent locomotion [42]. Yet while there are well-established frameworks for how wind affects flight costs (through airspeed and route selection) [34,43], there is no equivalent framework to estimate the metabolic consequences of kinematic variability. Nonetheless, turbulence may have widespread and significant implications for the costs of flapping flight, as a recent review showed that the wingbeat frequencies of 14 species of birds flying in the wild were highly variable [24,28].

This marked increase in kinematic variability seen in turbulent conditions may also help explain why the relationship between wingbeat frequency, amplitude and airspeed is so variable across species and studies [28]. Wingbeat frequency and amplitude are the two major kinematic determinants of aerodynamic power and should covary with parameters such as airspeed, yet a recent study found a striking lack of relationships across species [28]. Our results indicate that varying degrees of turbulence could introduce substantial noise into these relationships, potentially masking them entirely.

### 4.2. Behavioural responses to turbulence

In our trials, the main parameters influencing airspeed were the climb rate and HWC. This aligns with well-established frameworks that predict how flying animals should adjust their airspeed in relation to the costs of flight per unit distance flown [44–46]. The increase in airspeed due to turbulence was smaller than due to the wind, despite our study site being typified by low wind speeds (0.3–4.9, mean = 2.2 m s^−1^, estimated using ERA5 within the study period). Nonetheless, turbulence emerged as the primary driver of airspeed variability, with the standard deviation increasing around twofold from lowest to highest turbulence (23.9% of its range). Frequent changes in speed are usually more energetically demanding compared to maintaining a constant speed, because they require repeated accelerations [42,47], although in our case, some of the variability in airspeed may well be a passive response to part of the turbulence cascade, with birds being accelerated by gusts within turbulent eddies (cf. [18,48]).

The small increase in tortuosity with turbulence is unlikely to represent a substantive cost due to the scale of the tortuosity change that we observed (4.8% increase at maximum turbulence). Passive lateral displacements by turbulence may also explain some of this increase, although ultimately birds will have to respond to this variability to maintain a fixed course to the loft.

Changes in flight height were also predominantly driven by the HWC, although there was a small change in relation to turbulence. The question of how birds should adjust their route in relation to turbulence is not straightforward as it may vary depending on whether wind or convective turbulence dominates. The scale and strength of thermal updrafts increases with altitude in the lower half of the boundary layer (where the pigeons flew) [19]. By contrast, wind shear may be greater at lower altitudes, due to the interaction with complex topography and surface structures. Birds wishing to avoid turbulence may therefore increase or decrease their flight height, depending on the turbulence type. The decrease in flight height we observed is consistent with a strategy to reduce exposure to the strong convective turbulence at our study site, although the effect size was very small (1.7% of the altitude range). Turbulence did therefore not appear to be a primary driver of flight height, although we note our estimates of flight height will also be affected by the averaging of flight height within segments and the resolution of the DSM.

Overall, the way that birds responded to turbulence was fundamentally different to the way they responded to wind, as turbulence predicted the variation around the mean, for wingbeat frequency, amplitude and airspeed, whereas wind affected the mean airspeed and flight height. The main determinant of mean wingbeat frequency and amplitude was the climb rate. Researchers are increasingly using accelerometers to estimate flight effort (e.g. [28,49,50]), yet such studies focus on mean kinematic responses. Kinematic variability is seldom modelled (but see [24,28]) and while laboratory studies show the costs can be substantial, a framework predicting its metabolic consequences is currently lacking. Pigeons in this study only made small adjustments to their flight height in relation to turbulence, confirming that this response is primarily determined by wind. Nonetheless, a previous study found the same birds varied their horizontal route in a manner consistent with avoiding highly turbulent areas [18]. Horizontal route selection may be an effective way of avoiding turbulence, as both the shear- and thermally driven turbulence [18] vary with land cover and the interaction of wind with objects on the ground. Future improvements in the resolution of turbulence models or proxy development will hopefully enable behaviour to be modelled in relation to both types of turbulence for birds in the wild, as well as improve our understanding of the implications for flight power.

## Data Availability

The datasets generated and/or analysed during the current study along with the essential code employed for statistical modelling, including the final modelled dataset, are available from the Movebank Data Repository, https://doi.org/10.5441/001/1.284 (Lempidakis *et al.* 2023) [51]. Supplementary material is available online [52].
